# The HCMV‐encoded miR‐UL36‐3p promotes angiogenesis of endothelial cells by downregulating FOXO3

**DOI:** 10.1002/ame2.70196

**Published:** 2026-03-27

**Authors:** Chen Wang, Mengyu Li, Guosheng Li, Hao Zhang, Cheng Wang, Chen‐Yu Zhang

**Affiliations:** ^1^ School of Pharmacy Jiangsu University Zhenjiang Jiangsu China; ^2^ State Key Laboratory of Pharmaceutical Biotechnology, Chinese Academy of Medical Sciences Research Unit of Extracellular RNA, Nanjing Drum Tower Hospital Center of Molecular Diagnostic and Therapy, Jiangsu Engineering Research Center for MicroRNA Biology and Biotechnology, NJU Advanced Institute of Life Sciences (NAILS), Institute of Artificial Intelligence Biomedicine, School of Life Sciences Nanjing University Nanjing Jiangsu China; ^3^ Department of Critical Care Medicine Affiliated Jinling Hospital, Medical School of Nanjing University Nanjing Jiangsu China; ^4^ Department of Clinical Laboratory Nanjing Hospital of Chinese Medicine Affiliated to Nanjing University of Chinese Medicine Nanjing China

**Keywords:** angiogenesis, atherosclerosis, endothelial cell, FOXO3, HCMV, miRNA

## Abstract

**Background:**

Human cytomegalovirus (HCMV) infection is related to the acceleration of transplant vascular sclerosis, atherosclerosis, and coronary restenosis. A shared theme of these vascular illnesses is pathologic angiogenesis. Nevertheless, how HCMV infection causes angiogenesis is not fully understood. Human serum contains HCMV‐encoded miRNAs, and it is unclear whether these virus‐derived miRNAs can regulate angiogenesis. This research looks into HCMV‐encoded miRNA's role in angiogenesis of endothelial cells.

**Methods:**

Endothelial cell proliferation was examined by CCK8 assay, and cell migration capability was established using a Transwell Boyden Chamber. Western blotting alongside luciferase reporter assay verified the direct regulation of FOXO3 by HCMV‐encoded miRNAs, including hcmv‐miR‐UL36‐3p. hcmv‐miR‐UL36‐3p's pro‐angiogenic action was examined by angiogenesis assays (in vivo) and capillary tube formation (in vitro), which were performed by giving C57BL/6J mice subcutaneous Matrigel injections containing bFGF along with simultaneous injections of either hcmv‐miR‐UL36‐3p or ncRNA once every 4 days. After 8 days, Matrigel plugs were examined.

**Results:**

hcmv‐miR‐UL36‐3p was upregulated in patients with atherosclerosis. Overexpression of hcmv‐miR‐UL36‐3p enhanced capillary tube development, motility, and proliferation in endothelial cells. hcmv‐miR‐UL36‐3p promoted endothelial cell tube formation through directly binding to and downregulating FOXO3. Experiments in mice further confirmed that hcmv‐miR‐UL36‐3p promoted angiogenesis in vivo.

**Conclusions:**

The HCMV‐encoded miR‐UL36‐3p can trigger angiogenesis in endothelial cells by targeting FOXO3. Our work provides a conceivable mechanism of how HCMV‐encoded miRNAs contribute to vascular illness.

## Graphical abstract

This illustration shows the role of hcmv‐miR‐UL36‐3p in endothelial cell angiogenesis. During productive infection, endothelial cells express HCMV‐encoded miRNAs. Viral hcmv‐miR‐UL36‐3p promotes endothelial cell proliferation, migration, and capillary tube formation by directly targeting and downregulating FOXO3. The pro‐angiogenic activity of hcmv‐miR‐UL36‐3p is validated in a mouse model: C57BL/6J mice were injected subcutaneously with Matrigel and injected via tail vein with hcmv‐miR‐UL36‐3p Agomir twice; the Matrigel plugs were subsequently analyzed, revealing enhanced angiogenesis.

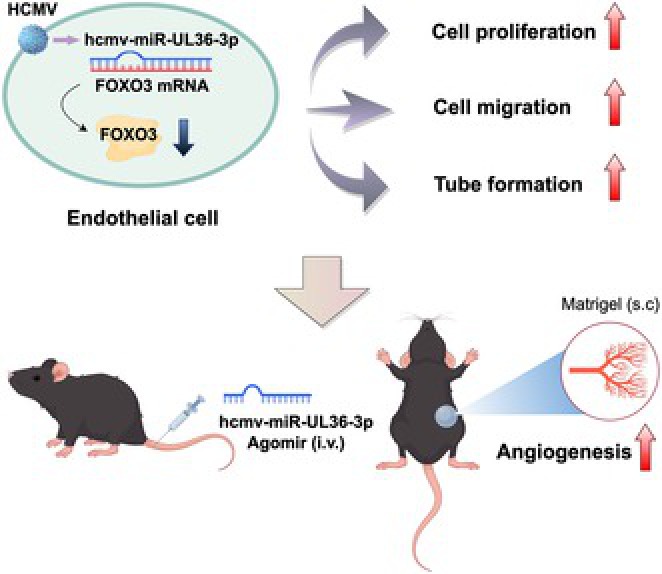

## 1 INTRODUCTION

The human cytomegalovirus, or HCMV, is a common herpesvirus that may affect as much as 90% of adults and lasts for the host's lifetime. Infection with HCMV can alter the host immune system and affect how inflammatory and cancerous conditions develop.^1^, ^2^, ^3^ Clinical evidence and systematic meta‐analyses have revealed a significant relationship between HCMV infection and vascular diseases, including the acceleration of atherosclerosis, transplant vascular sclerosis, and coronary restenosis.^4^, ^5^, ^6^, ^7^, ^8^ HCMV infection is a possible contributor to vascular illness, especially in the Asian population.^4^

The proliferation, migration, and morphogenesis of endothelial cells influence the intricate biological process known as angiogenesis. Although angiogenesis is a normal physiological event, many vascular disorders, such as plaque progression in atherosclerotic patients, have been linked to its dysregulation.^9^ Plaque rupture may specifically result from the development of fresh blood vessels inside the plaque.^10^, ^11^, ^12^ HCMV‐exacerbated vascular illness is believed to be caused by the angiogenic response that is induced when HCMV infects endothelial cells.^13^, ^14^ Despite extensive research, the precise mechanism by which HCMV infection induces angiogenesis remains incompletely elucidated.

Identifying HCMV‐encoded microRNAs (miRNAs) has paved the way for additional research in the last few years. A minimum of 24 miRNAs targeting genes that control viral growth as well as immune evasion are expressed by HCMV during active infection.^15^ These virus‐derived miRNAs have been detected in human serum. For instance, several HCMV‐encoded miRNAs have been identified in serum from people with high risk HCMV infection, or with fever of unknown origin (FUO)^16^, ^17^; an elevated essential hypertension risk was separately linked to hcmv‐miR‐UL112 levels.^18^

Understanding how HCMV miRNAs affect angiogenesis may offer novel perceptions about the pathogenesis of HCMV‐related vascular illnesses and potentially generate novel treatment approaches. Here, we studied HCMV miRNAs' regulatory effects on angiogenesis in endothelial cells. We demonstrate that, by targeting FOXO3, hcmv‐miR‐UL36‐3p promotes proliferation, migration, and tube formation of endothelial cells and promotes angiogenesis in vivo.

## 2 METHODS

### 2.1 Cell culture

Shanghai's Cell Bank, Chinese Academy of Sciences (China) supplied the HMEC‐1 cell line, which was cultured in Gibco's MCDB‐131 medium (10372019) containing Gibco's MVGS (S00525) and 10% FBS.

### 2.2 Sample collection

The present work has been accepted by Jinling Hospital's ethics committee board (approval number 2022DZGZR‐068) and conforms with the World Medical Association Declaration of Helsinki. Everyone who took part provided informed consent in writing. Written informed consent was acquired from the legally appointed person in place of the individual taking part for patients whose ability to authorize was impaired. We investigated serum HCMV miRNA expression in healthy controls and patients with atherosclerosis (*n* = 24).

### 2.3 Animals

Jiangsu University's Laboratory Animal Research Center in Zhenjiang, China, supplied 8‐week‐old C57BL/6J male mice, which were kept in an environment free of pathogens. Jiangsu University's Animal Care and Use Committee gave its approval to all animal experiments (UJS‐ACUC‐2025092801). Randomization and blinding were rigorously implemented during treatment and outcome assessment.

### 2.4 RNA isolation and quantitative RT‐PCR

TRIzol Reagent (Invitrogen, Shanghai, China) was used to isolate total cellular RNA. We collected total RNA from serum using phenol/chloroform purification and precipitated it using isopropyl alcohol. Quantitative RT‐PCR (qRT‐PCR) was carried out on Roche Diagnostics' LightCycler® 480 II PCR System (Mannheim, Germany) utilizing Applied Biosystems' TaqMan miRNA probes (Foster City, CA, USA). Every reaction was carried out in triplicate, along with the no‐template controls. Fixed threshold parameters were used to calculate the Ct values. Cellular miRNA expression levels were normalized to U6 snRNA. qRT‐PCR was also used to create a standard curve for a set of synthetic miRNA oligonucleotides at given doses. Absolute serum/plasma concentrations of each miRNA were quantified using the standard curve as a guide.

### 2.5 Overexpression of HCMV miRNAs

Invitrogen (Shanghai, China) provided scrambled negative control RNA (ncRNA) and synthetic hcmv‐miR‐UL59, hcmv‐miR‐UL36‐3p, and hcmv‐miR‐US25‐2‐3p. Equivalent hcmv‐miR‐UL59, hcmv‐miR‐UL36‐3p, hcmv‐miR‐US25‐2‐3p amounts (50 nM) or ncRNA were transfected using Lipofectamine RNAiMAX (Invitrogen) following the manufacturer's directions. Guangzhou's RiboBio (China) supplied MiRNA Agomir.

GenePharma (Shanghai, China) designed and produced three siRNA variants that target distinct sites in human FOXO3. The negative control was scrambled siRNA (ncRNA). Lipofectamine RNAiMAX (Invitrogen) helped transfect siRNAs (50 nM) into HMEC‐1 cells.

Table S2 lists FOXO3‐targeting siRNA as well as HCMV miRNA sequences.

### 2.6 Cell Counting Kit‐8 assay

Cell Counting Kit‐8 (CCK‐8, Dojindo) was used as directed by the manufacturer to assess HMEC‐1 cell viability. After transfection of HCMV miRNAs, HMEC‐1 cells were plated in 96‐well plates at a density of 5.0 × 10^3^ cells/well. They underwent incubation in MCDB‐131 medium containing 10% FBS as well as MVGS. Ten microliters of CCK‐8 liquid were introduced to the well at various intervals and allowed to incubate for 3 h. At 450 nm, absorbance (*A*) was measured.

### 2.7 Cell migration assay

MCDB‐131 culture medium without serum was used to suspend HMEC‐1 cells. Next, 500 μL of MCDB‐131 with 10% FBS was added to the lower chamber of a Transwell Boyden Chamber (6.5 mm, Costar), and a 100‐μL cell suspension (4 × 10^4^ cells/well) was added to the upper chamber. After incubating for 12 h, non‐migrant cells found on the filter membrane's topmost layer were carefully scraped off using a cotton swab. After passage through a polycarbonate membrane covered with 0.1% gelatin matrix (8‐μm pore size), cells were fixed at room temperature for 15 min using 4% paraformaldehyde. After three rounds of washing with distilled water, the membrane was dyed for 15 min with 0.1% crystal violet. A BX51 photomicroscope (Olympus, Japan) allowed was used to take pictures of the migrating cells. Blind counting of the migrated cells on the membrane's lower surface—five fields in each chamber—enabled measurement of cell migration.

### 2.8 Apoptosis assay

After culturing for 18 h in serum‐free media following ncRNA, hcmv‐miR‐UL59, or hcmv‐miR‐UL36‐3p transfection, HMEC‐1 cells were isolated to examine apoptosis. The cells were suspended again in 1× binding buffer at 1 × 10^6^ cells/mL after being twice rinsed with cold PBS in accordance with directions given by the manufacturer for the FITC‐Annexin V Apoptosis Detection Kit I (BD Biosciences). After transferring 100 μL of cell suspension to a culture tube (5 mL), PI and FITC‐Annexin V were added. Within an hour of dyeing, the cells were examined using flow cytometry (BD Biosciences) after being incubated in the dark at room temperature for 15 min. The bottom right quadrant contains a biparametric histogram showing cells in the early apoptotic stage Figure 2E. The upper right quadrant shows cells in the late apoptotic stage. The bottom left quadrant shows double‐negative viable cells.

### 2.9 Angiogenesis in vitro (capillary tube formation assay)

The test for in vitro endothelial tube creation was carried out as previously described.^19^, ^20^ Each well of a 24‐well plate received 200 μL of Matrigel (BD Bioscience), which was then left to polymerize for 1 h at 37°C. After ncRNA, hcmv‐miR‐UL59, hcmv‐miR‐UL36‐3p, and hcmv‐miR‐US25‐2‐3p transfection, we suspended HMEC‐1 cells in MCDB‐131 medium without FBS and seeded them at a density of 1 × 10^5^ cells/well. After incubating for 6 h at 37°C, a light microscope examined the cells to determine whether capillary‐like structures had formed. The meshes of formed tubes, which serve as an indicator of in vitro angiogenesis, were quantified across three fields.

### 2.10 Angiogenesis in vivo (Matrigel plug assay)

Matrigel is a soluble extract of the Engelbreth‐Holm‐Swarm tumor's basement membrane. Endothelial cells may migrate into the Matrigel plug and create capillaries when Matrigel is injected subcutaneously. This angiogenic cycle can be accelerated by mixing the Matrigel with recognized angiogenic growth factors, such as basic fibroblast growth factor (bFGF). Erythrocytes are present in the freshly created vessels, suggesting their role as functional capillaries.^19^, ^20^

In vivo Matrigel plug experiments were carried out.^21^ Briefly, 150 ng of bFGF (Sigma) and 40 units of heparin (Sigma) were combined with 500 μL of growth factor‐reduced Matrigel (BD Bioscience). C57BL/6J mice were randomly divided into two groups (*n* = 6 per group) and given a subcutaneous injection of the combination in the middle of their abdomens. In vivo, the injected Matrigel quickly solidified into a gel that lasted for no less than 10 days. Beginning on the day we injected the Matrigel, the mice were given intravenous injections of 10 nmol of either ncRNA Agomir or hcmv‐miR‐UL36‐3p Agomir every 4 days. After 8 days, the mice were euthanized, and the Matrigel plugs were excised and captured on camera. Then, the plugs were fixed in 4% paraformaldehyde, embedded in paraffin, and sectioned. Hematoxylin and eosin (H&E) stain was added to the slices. To perform a quantitative analysis, the number of erythrocyte‐filled blood vessels in the Matrigel (capillary density) was counted in five independent fields from three sections.

### 2.11 HCMV miRNA target prediction

Target gene prediction for HCMV miRNAs was conducted using TargetScan Human 5.2 Custom (https://www.targetscan.org/vert_50/seedmatch.html).^22^

### 2.12 Plasmid construction and luciferase assay

Invitrogen produced a partial human FOXO3 3′‐UTR sequence that contains the anticipated HCMV miRNA binding sites. Two single chains were annealed at 60°C to create a double‐stranded molecule, which was then introduced into p‐MIR‐report plasmid (Ambion). Sequencing verified successful insertion. We cultivated the cells in six‐well plates for the luciferase reporter tests. Using Lipofectamine 2000 (Invitrogen), equivalent amounts of ncRNA or HCMV miRNAs were transfected into every well, along with 2 μg each of β‐galactosidase expression vector (Ambion) and firefly luciferase reporter plasmid containing WT FOXO3 3′‐UTR or a mutant reporter plasmid (Mut). The transfection control was the β‐galactosidase vector. Twenty‐four hours after transfection, we used luciferase assay kits (Promega, Madison, WI, USA) to examine the cells. No fewer than three separate experiments are represented in the published data.

### 2.13 Western blotting

SDS polyacrylamide gels were used to separate protein samples, which were wet electrophoretically transferred onto PVDF membranes (Millipore). Then, 5% nonfat dry milk in Tris‐buffered saline (TBS)/0.1% Tween was added to block the blots at room temperature for 1 h. Anti‐GAPDH (MA5–15738, Thermo Fisher Scientific) and anti‐FOXO3 (2497, Cell Signaling Technology) (both primary antibodies) were then added and incubated at 4°C for the whole night. Blots were created using Pierce's chemiluminescent reagents after incubating with secondary antibodies conjugated with HRP. The loading control was GAPDH. ImageJ software was used to carry out quantification.

### 2.14 Statistical analysis

GraphPad Prism v9 was used to carry out data analyses. Information is shown as either the mean ± standard deviation (SD) or the mean ± standard error of mean (SEM). Two sets of data were compared using two‐tailed Student's *t*‐tests or a Mann–Whitney test. Experiments with several groups were carried out using one‐way ANOVA, whereas those with two distinct variables were carried out using two‐way ANOVA. *n* denotes biological replicates. A *p*‐value < 0.05 suggested significance.

## 3 RESULTS

### 3.1 hcmv‐miR‐UL36‐3p is upregulated in patients with atherosclerosis

**FIGURE 1:**
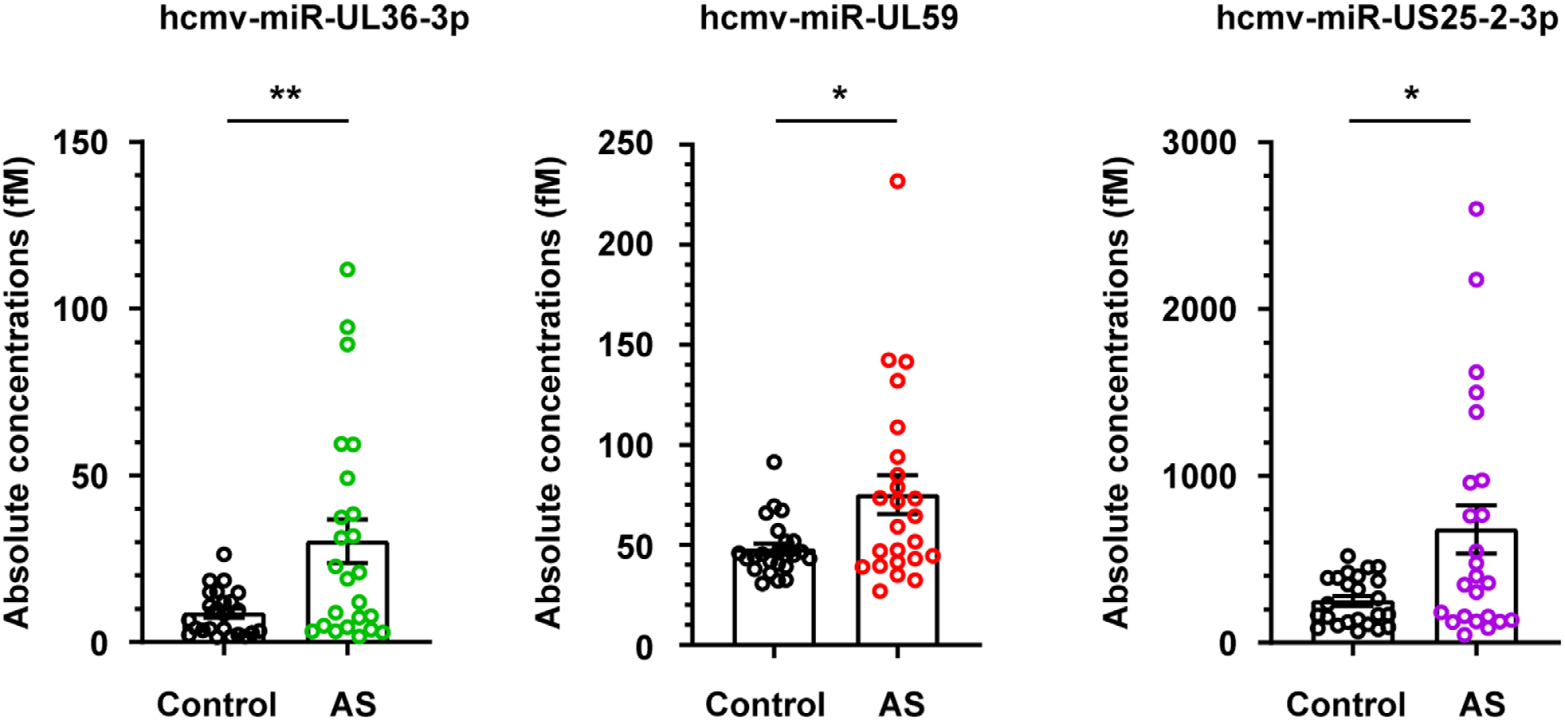
Absolute concentrations of HCMV‐miRNAs in the serum of atherosclerosis patients (AS) and healthy controls (Control) (*n* = 24) were determined by qRT‐PCR. Data are expressed as means ± SEM. The non‐parametric Mann–Whitney test shows significance. *p < 0.05, **p < 0.01.

The aberrant HCMV miRNA serum profile in patients with fever has been explored in a multiphase case–control study.^17^ Here, we examined expression patterns of several candidate HCMV miRNAs in patients with atherosclerosis (AS). qRT‐PCR analysis revealed that hcmv‐miR‐UL36‐3p, hcmv‐miR‐UL59, and hcmv‐miR‐US25‐2‐3p in the serum were significantly upregulated in AS patients compared with healthy controls (Figure 1).

### 3.2 hcmv‐miR‐UL36‐3p enhances angiogenesis by enabling endothelial cell migration and capillary tube formation

**FIGURE 2:**
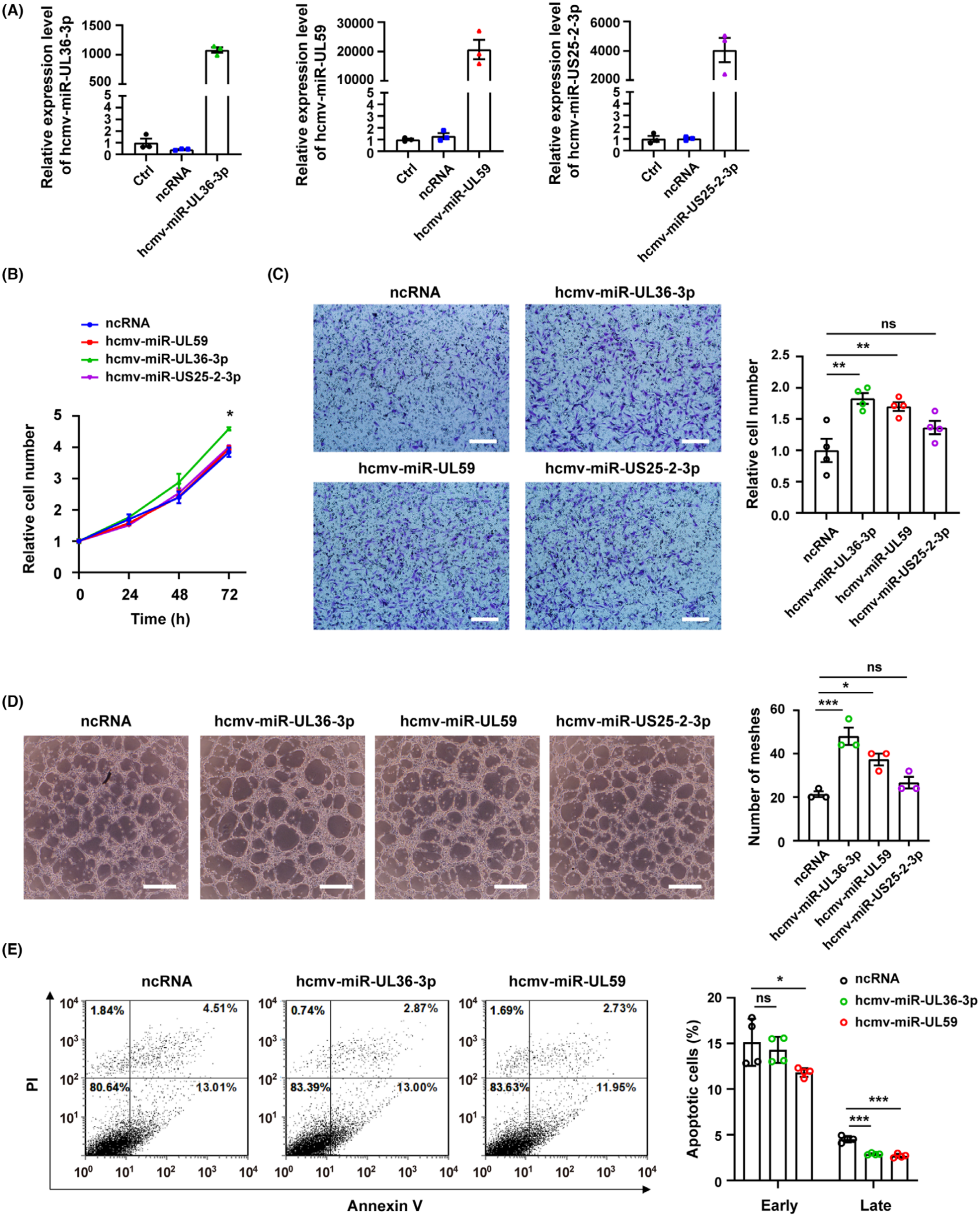
HCMV‐miRNAs promote endothelial cell function. (A) Overexpression of HCMV miRNAs in HMEC‐1 cells (*n* = 3). (B) Cell viability test at 0, 24, 48, and 72 h after transfecting HMEC‐1 cells with equivalent HCMV miRNA or ncRNA doses. The asterisks indicate significant differences between hcmv‐miR‐UL36‐3p and ncRNA. (C) Transwell examination of HMEC‐1 cells that were given equivalent ncRNA or HCMV miRNA doses. Left panel shows representative images, and right panel shows the data analysis (*n* = 4). Scale bars, 100 μm. (D) Typical pictures of HMEC‐1 cells on Matrigel that were transfected with different HCMV miRNAs. Scale bars, 100 μm. Right: Quantifying numbers of newly formed vessels in Matrigel sections (*n* = 3). (E) Flow cytometry analyzes cell apoptosis profiles. Right panel shows data analysis (*n* = 4). All data expressed as means ± SEM (A–D) or means ± SD (E). Two‐way ANOVA along with Dunnett's post hoc test (B) and one‐way ANOVA along with Dunnett's post hoc test (C–E) determined significance. *p < 0.05, **p < 0.01, ***p < 0.001. ns, not significant.

Next, we studied the role of these three HCMV miRNAs in angiogenesis. HMEC‐1 cells were transfected with hcmv‐miR‐UL36‐3p, hcmv‐miR‐UL59, or hcmv‐miR‐US25‐2‐3p. This helped determine their role in endothelial cells. Efficient overexpression of the HCMV miRNAs was confirmed by qRT‐PCR (Figure 2A). The Cell Counting Kit‐8 helped determine HMEC‐1 proliferation rates. Compared with cells that were given ncRNA, those that were given hcmv‐miR‐UL36‐3p showed higher proliferation rates (Figure 2B), while hcmv‐miR‐UL59 and hcmv‐miR‐US25‐2‐3p did not affect endothelial cell growth. Following this, we carried out the transwell assay to evaluate HCMV miRNAs' function in cell migration. HMEC‐1 cells that were given hcmv‐miR‐UL36‐3p or hcmv‐miR‐UL59 showed a higher migration rate than cells that were given ncRNA, as seen in Figure 2C. In contrast, the impact of hcmv‐miR‐US25‐2‐3p was weak.

Endothelial cell migration from preexisting blood arteries to create new capillaries in response to stimuli is a crucial angiogenesis stage.^23^ We carried out an in vitro test to explore capillary‐like tube forms, which mimic microvascular networks on Matrigel; it helped us determine how HCMV miRNAs affect angiogenic morphogenesis.^19^, ^20^ Following the protocol described above, capillary‐like forms were created by HMEC‐1 cells. Elevated exogenous HCMV miRNAs advanced tube formation (Figure 2D). Among them, hcmv‐miR‐UL36‐3p had the most significant effect, hcmv‐miR‐UL59 had a strong effect, and hcmv‐miR‐US25‐2‐3p had a mild effect.

We also estimated cell apoptosis after HCMV miRNA overexpression in HMEC‐1 cells. hcmv‐miR‐UL59‐transfected cells had a lower percentage of early apoptotic cells, as seen in flow analysis, and late apoptotic cells were significantly fewer in both HCMV miRNA‐treated cells, suggesting that hcmv‐miR‐UL59 as well as hcmv‐miR‐UL36‐3p could inhibit endothelial cell apoptosis (Figure 2E).

### 3.3 hcmv‐miR‐UL36‐3p directly targets FOXO3

**FIGURE 3:**
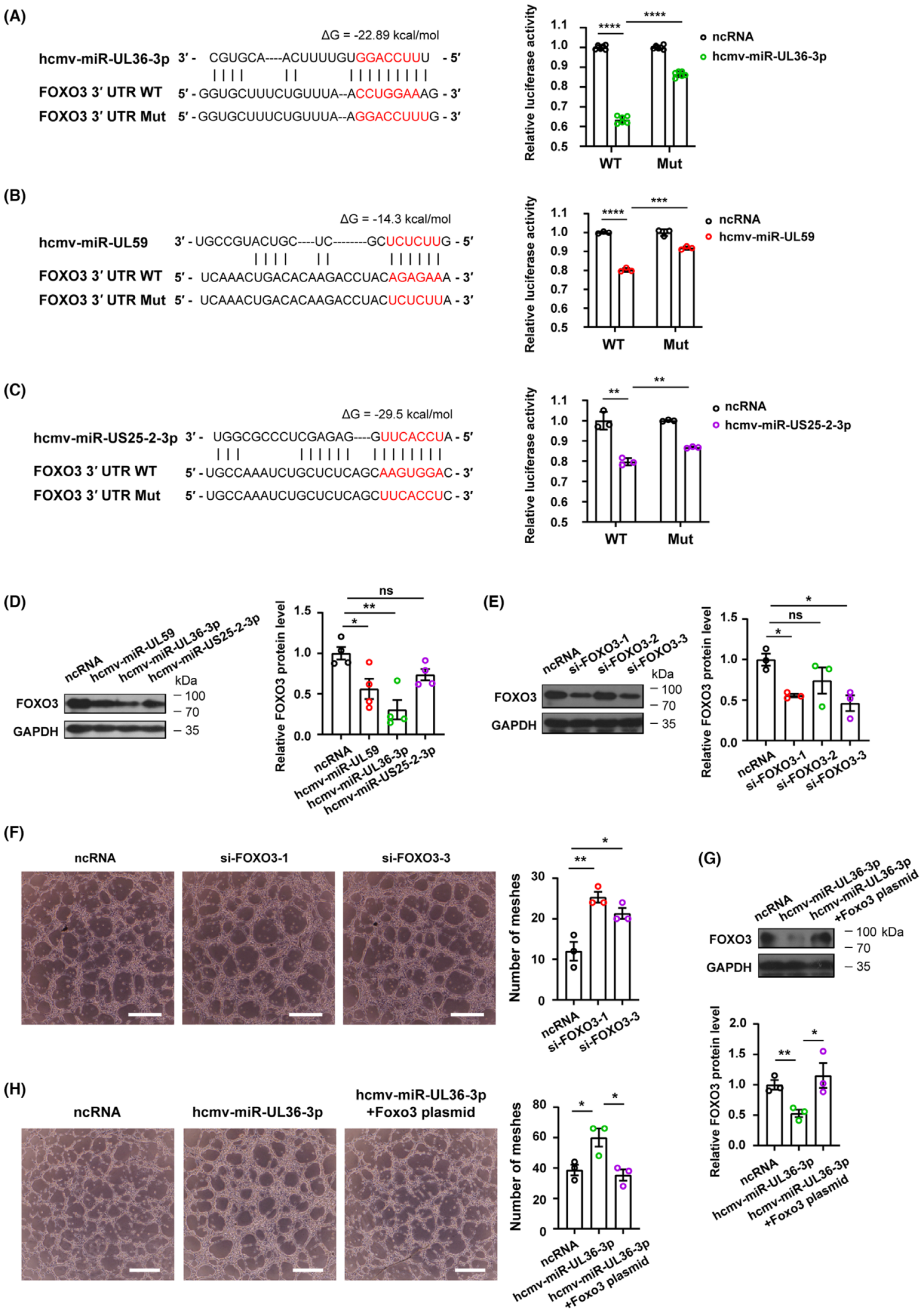
HCMV‐miRNAs directly regulate human FOXO3 expression. (A–C) Detecting human FOXO3 3′‐UTR by HCMV miRNAs: Hcmv‐miR‐UL36‐3p (A, *n* = 6), hcmv‐miR‐UL59 (B, *n* = 3), hcmv‐miR‐US25‐2‐3p (C, *n* = 3). Left panel, mutant (Mut) or wild‐type (WT) miRNA binding sites (the portion interacting with the miRNA seed region was mutated) in the FOXO3 3′‐UTR. Right panel, the data of firefly luciferase are shown as relative luciferase activity after being adjusted to β‐galactosidase activity. Luciferase activity in cells transfected with ncRNA was adjusted to 1 for comparison. (D) Western blotting of human FOXO3 protein in HCMV miRNA‐treated HMEC‐1 cells. The loading control is GAPDH. Right: Quantifying normalized expression (*n* = 4). (E) Western blotting of FOXO3 protein in HMEC‐1 cells 24 h after transfection with three siRNAs against FOXO3 (si‐FOXO3‐1, 2, 3) or a scrambled siRNA negative control (ncRNA). Right: Quantifying normalized expression levels (*n* = 3). (F) Typical Matrigel pictures of HMEC‐1 cells containing equivalent ncRNA or si‐FOXO3 doses from three independent experiments are shown. Scale bars, 100 μm. Right: Quantifying numbers of newly formed vessels in Matrigel sections (*n* = 3). (G) Western blotting of FOXO3 protein in HMEC‐1 cells transfected with ncRNA, hcmv‐miR‐UL36‐3p, or hcmv‐miR‐UL36‐3p plus FOXO3 overexpression plasmid. Lower panel, quantifying normalized expression levels (*n* = 3). (H) Typical Matrigel pictures of HMEC‐1 cells containing ncRNA, hcmv‐miR‐UL36‐3p, or hcmv‐miR‐UL36‐3p plus FOXO3 overexpression plasmid. Right, quantifying numbers of newly formed vessels in Matrigel sections (*n* = 3). All data expressed as means ± SD (A–C) or means ± SEM (D–H). Two‐tailed Student's *t*‐test (A–C) and one‐way ANOVA with Dunnett's post hoc test (D–H) show significance. *p < 0.05, **p < 0.01, ***p < 0.001, ****p < 0.0001. ns, not significant.

TargetScan Human 5.2 Custom assisted with target gene prediction that helped us investigate the three HCMV miRNA targets. The analysis revealed that Forkhead box O3 (FOXO3) is a common target of all three HCMV miRNAs (Figure 3A–C, left panel). In developed endothelial cells, FOXO3 is a prevalent Foxo isoform that prevents cell migration and tube formation. The control of adult vessel development is significantly influenced by this transcription factor.^24^

Luciferase reporter assays established the direct binding of these HCMV miRNAs to the supposed 3′‐UTR sites of human FOXO3 mRNA. In the reporter plasmid, we cloned a FOXO3 3′‐UTR segment with the projected miRNA binding sites downstream of the firefly luciferase gene. This wild‐type construct (WT) was co‐transfected with a β‐galactosidase‐expressing control plasmid as well as a specific HCMV miRNA or ncRNA. Compared with the ncRNA control, overexpression of hcmv‐miR‐UL36‐3p, hcmv‐miR‐UL59, or hcmv‐miR‐US25‐2‐3p significantly lowered luciferase activity, standardized to β‐galactosidase activity (Figure 3A–C, right panel). Furthermore, potential FOXO3 3′‐UTR binding sites were mutated into complementary sequences to generate a mutant reporter plasmid (Mut). These mutations partially restored luciferase reporter activity that had been suppressed by HCMV miRNA expression. The miRNA‐mRNA interactions are greatly affected by the binding sites that have been found.

Furthermore, we examined whether three HCMV miRNAs could regulate FOXO3 protein expression. As expected, in HMEC‐1 cells, FOXO3 protein expression was markedly downregulated after hcmv‐miR‐UL36‐3p overexpression. hcmv‐miR‐UL59 also significantly reduced FOXO3 protein level, whereas hcmv‐miR‐US25‐2‐3p exhibited only a weak effect (Figure 3D). In summary, these results identify hcmv‐miR‐UL36‐3p as the dominant negative regulator of FOXO3 among the three HCMV miRNAs—acting via direct binding to the FOXO3 3′‐UTR—and establish its functional primacy in driving endothelial cell proliferation, migration, and tube formation.

Lastly, siRNAs targeting FOXO3 were administered to HMEC‐1 cells, and tube formation was evaluated. Compared to the control siRNA, effective FOXO3 expression interference was achieved by si‐FOXO3‐1 and si‐FOXO3‐3 (Figure 3E). In line with previous findings, in HMEC‐1 cells, FOXO3 knockdown brought on by siRNA raised tube formation (Figure 3F). We next investigated whether FOXO3 overexpression could rescue the hcmv‐miR‐UL36‐3p‐mediated suppression of FOXO3 expression and its downstream functional consequences in HMEC‐1 cells. To this end, we constructed a FOXO3 expression plasmid encoding the full‐length open reading frame (ORF) but lacking the hcmv‐miR‐UL36‐3p‐responsive 3′‐UTR, thereby rendering FOXO3 resistant to miRNA‐dependent repression. Co‐transfection of this plasmid with hcmv‐miR‐UL36‐3p significantly restored FOXO3 protein levels relative to cells transfected with hcmv‐miR‐UL36‐3p alone (Figure 3G), confirming effective rescue of FOXO3 suppression. Consistently, FOXO3 overexpression reversed hcmv‐miR‐UL36‐3p‐induced tube formation in HMEC‐1 cells (Figure 3H). These data confirm the anti‐angiogenic activity of the FOXO3 protein and suggest that hcmv‐miR‐UL36‐3p may facilitate angiogenesis by reducing FOXO3 protein levels.

### 3.4 hcmv‐miR‐UL36‐3p promotes angiogenesis in vivo

**FIGURE 4:**
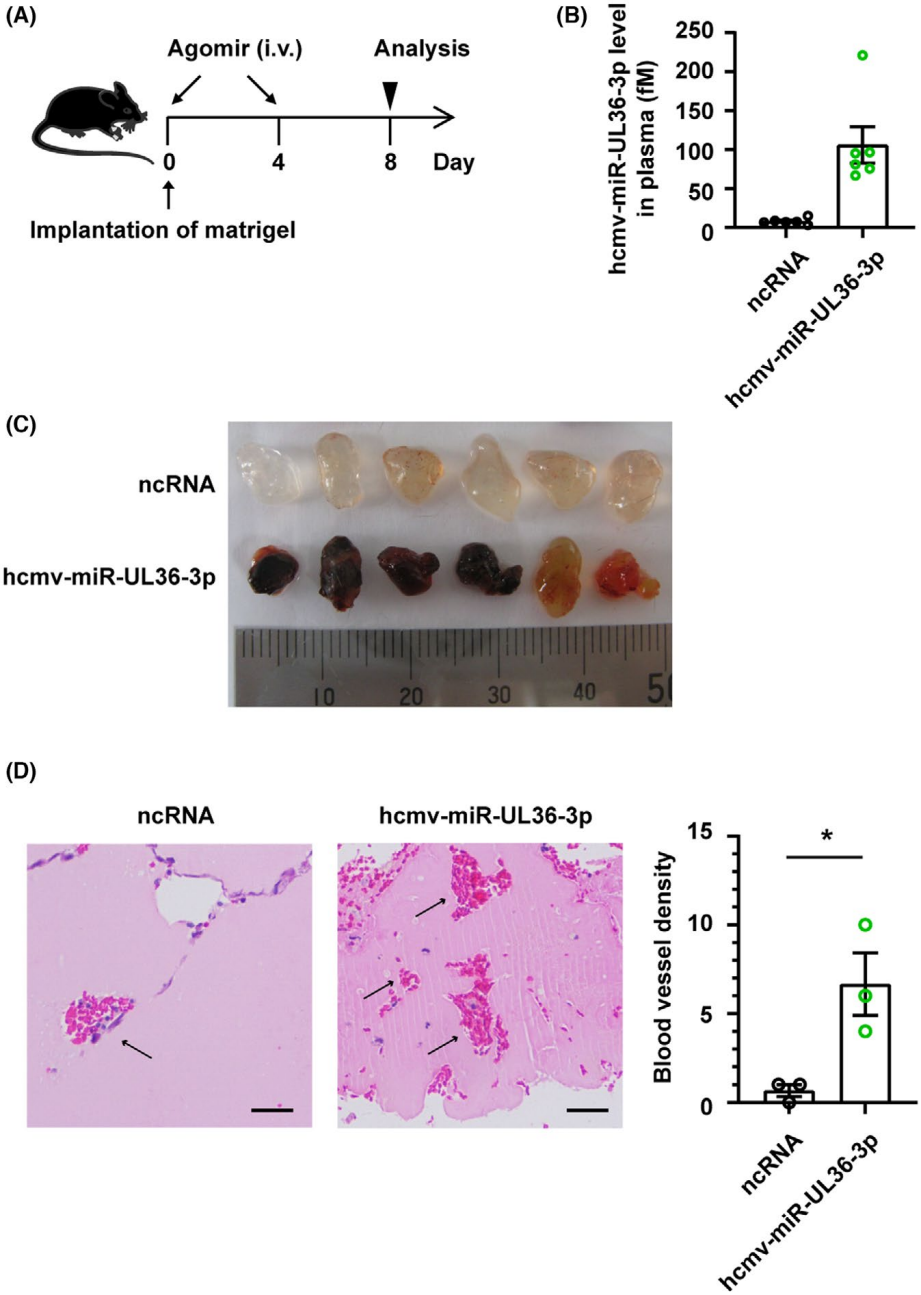
hcmv‐miR‐UL36‐3p promotes angiogenesis in vivo. (A) Experimental plan of the in vivo Matrigel plug assay. (B) qRT‐PCR analysis of hcmv‐miR‐UL36‐3p plasma levels in mice containing ncRNA or hcmv‐miR‐UL36‐3p Agomir (*n* = 6). (C) Matrigel plug morphology (*n* = 6). (D) Matrigel plugs (panel C) showing sections stained with H&E, with arrows indicating the luminal assemblies comprising red blood cells. Scale bar, 20 μm. Right: Quantifying numbers of newly formed vessels in Matrigel sections (*n* = 3). Data are expressed as means ± SEM. Two‐tailed Student's *t*‐test show significance. *p < 0.05.

Matrigel plug assays helped us explore the capability of hcmv‐miR‐UL36‐3p to stimulate in vivo angiogenesis.^19^, ^20^ Figure 4A shows the experimental design. Compared to those treated with ncRNA Agomir, mice intravenously injected with hcmv‐miR‐UL36‐3p Agomir exhibited significantly increased plasma levels of hcmv‐miR‐UL36‐3p—to 100 fM, a concentration within the clinically observed range (Figure 4B). The Matrigel plugs retrieved from ncRNA‐treated mice appeared pale pink, whereas those from hcmv‐miR‐UL36‐3p‐treated mice displayed a dark red color (Figure 4C), suggesting extensive formation of new blood vessels within the plugs. Histological analysis further revealed that the Matrigel plugs from hcmv‐miR‐UL36‐3p‐injected mice contained numerous erythrocyte‐filled blood vessels (Figure 4D).

Collectively, our findings suggest that during HCMV infection, the virus‐encoded hcmv‐miR‐UL36‐3p is upregulated in the serum of patients with atherosclerosis and may promote angiogenesis by directly targeting FOXO3, thereby contributing to the pathogenesis of HCMV‐associated vascular disease.

## 4 DISCUSSION

Numerous vascular illnesses involving pathological angiogenesis have been linked to HCMV. For example, HCMV‐infected people with atherosclerosis are often at risk of coronary heart disease, particularly in the context of an aging population.^25^ This study identifies hcmv‐miR‐UL36‐3p as a pro‐angiogenic viral effector, providing a mechanistic link to the pathogenesis of HCMV‐associated vascular disorders.

Beyond the well‐documented dysregulation of host miRNAs during infection, an emerging paradigm in viral pathogenesis is the direct deployment of virally encoded miRNAs as precise tools to hijack host cellular processes. Herpesviruses exemplify this strategy: miRNAs from Kaposi's sarcoma‐associated herpesvirus (KSHV) and human herpesvirus 6A (HHV‐6A) directly modulate host cell migration^26^ and immune evasion pathways.^27^ Our discovery that HCMV encodes hcmv‐miR‐UL36‐3p as a potent pro‐angiogenic factor extends this paradigm into the realm of vascular biology. It suggests that HCMV, like its herpesvirus relatives, may employ endogenous non‐coding RNAs not merely as byproducts of infection, but as functional effectors to remodel the host vasculature.

HCMV establishes lifelong persistence in endothelial cells, which may lead to chronic activation and sustained dysregulation of endothelial function as a pathogenic consequence.^28^ Previous studies have shown that HCMV induces angiogenesis through multiple pathways: direct viral binding to the epidermal growth factor receptor and integrins^13^; a pro‐angiogenic, virus‐free endothelial secretome enriched in cytokines—including granulocyte macrophage colony‐stimulating factor (GM‐CSF), interleukin‐6 (IL‐6), and IL‐8/CXCL8^29^—as well as virally encoded proteins such as the CEACAM1‐like molecule pUL7^30^ and the chemokine receptor US28.^31^, ^32^ HCMV infection also induces upregulation of host miR‐138, miR‐217, and miR‐199a‐5p in endothelial cells that inhibit sirtuin 1 (SIRT1), thus augmenting angiogenesis.^33^, ^34^ These findings reveal the host's adaptive or maladaptive response to viral infection. In contrast, hcmv‐miR‐UL36‐3p is a virally encoded miRNA whose expression is tightly coupled to the HCMV lytic replication cycle, reflecting an active, sequence‐specific viral strategy to reprogram host physiology. Unlike immunogenic viral proteins, hcmv‐miR‐UL36‐3p mediates persistent and stealthy modulation of host gene expression via post‐transcriptional repression, enabling precise, fine‐tuning of endothelial signaling networks.

The pro‐angiogenic activity of hcmv‐miR‐UL36‐3p has clinical relevance. During productive infection, endothelial cells express HCMV‐encoded miRNAs^35^; hcmv‐miR‐US25‐1, hcmv‐miR‐US25‐2‐3p, and hcmv‐miR‐UL36‐3p are highly upregulated as demonstrated by AGO‐CLIP‐seq as well as small RNA‐seq.^36^, ^37^ hcmv‐miR‐UL36 has been shown to downregulate UL138, a latency‐associated protein, thereby promoting HCMV replication.^38^, ^39^ Here, we demonstrate that hcmv‐miR‐UL36‐3p promotes angiogenesis in vitro as well as in vivo, highlighting its functional impact on the host. Notably, the serum concentration of hcmv‐miR‐UL36‐3p in AS patients ranges from 30 to 100 fM. In our mouse model, circulating level of hcmv‐miR‐UL36‐3p reached 100 fM, which falls within the clinically observed physiological range. At this level, hcmv‐miR‐UL36‐3p exhibited efficient pro‐angiogenic activity, suggesting that it may have functional significance in individuals with HCMV infection.

The pro‐angiogenic effect of hcmv‐miR‐UL36‐3p is mediated through direct repression of FOXO3—a transcriptional repressor of endothelial nitric oxide synthase (eNOS) and a potent inducer of endothelial apoptosis.^24^, ^40^ FOXO3 inhibition results in eNOS upregulation and enhanced nitric oxide (NO) production, which is essential for neovascularization.^41^ Therefore, we speculate that hcmv‐miR‐UL36‐3p might act via the FOXO3‐eNOS signaling axis. In parallel, the cyclic heptapeptide FZ1 was recently identified as an integrin αvβ3 agonist that promotes angiogenesis and facilitates diabetic skin wound healing by activating the FAK‐AKT/ERK‐VEGFC pathway.^42^ Such diverse signaling inputs converge on endothelial cell functions, underscoring the complexity of angiogenic regulation. In addition to FOXO3, other potential targets of hcmv‐miR‐UL36‐3p have been predicted by TargetScan (Table S1). Gene Ontology (GO) enrichment analysis of the top 100 targets—performed by Metascape (https://metascape.org)—revealed significant enrichment for “negative regulation of cell migration” (Figure S1), consistent with our experimental observation of enhanced endothelial cell migration and angiogenesis induced by hcmv‐miR‐UL36‐3p.

This study identifies hcmv‐miR‐UL36‐3p as a novel therapeutic target for HCMV‐associated cardiovascular diseases. Cardiovascular events in these patients likely arise from both direct vascular inflammation and accelerated atherosclerosis.^43^, ^44^ Atherosclerotic vulnerable plaques are prone to rupture and bleeding, which are closely related to the onset of cardiovascular events. The formation of these plaques involves two key factors: First, macrophage foaming that softens the plaque; second, extensive intraplaque neovascularization, leading to bleeding and thrombosis.^45^, ^46^ Based on these findings, HCMV‐encoded miRNAs might result in clinical events in patients with HCMV‐associated AS by promoting angiogenesis. Besides control of systemic inflammation through approaches such as polyphenol‐based interventions,^47^ and traditional cardiovascular risk management to improve clinical outcomes, targeting HCMV miRNAs might suggest a pathogen‐directed, precision therapeutic strategy.

Several limitations remain to be addressed. Transcriptomic or proteomic profiling would broaden our understanding of hcmv‐miR‐UL36‐3p's regulatory network beyond FOXO3. Antagomir‐mediated inhibition of hcmv‐miR‐UL36‐3p would rigorously validate on‐target specificity. Generating an HCMV mutant deficient in hcmv‐miR‐UL36‐3p and using it to infect mice would enable direct assessment of this miRNA's contribution to virus‐induced angiogenesis.

## 5 CONCLUSION

HCMV‐encoded hcmv‐miR‐UL36‐3p is significantly upregulated in AS patients. hcmv‐miR‐UL36‐3p promotes angiogenesis of endothelial cells both in vitro and in vivo by directly targeting FOXO3, suggesting a potential pathogenic role in HCMV‐associated vascular dysfunction.

## AUTHOR CONTRIBUTIONS

**Chen Wang:** Conceptualization; data curation; formal analysis; funding acquisition; writing – original draft; writing – review and editing. **Mengyu Li:** Data curation. **Guosheng Li:** Data curation. **Hao Zhang:** Data curation. **Cheng Wang:** Data curation; formal analysis; methodology; resources. **Chen‐Yu Zhang:** Project administration.

## FUNDING INFORMATION

Our research was supported by endowments from the Senior Talent Foundation of Jiangsu University (5501290013).

## CONFLICT OF INTEREST STATEMENT

Authors declare no competing interests.

## ETHICS STATEMENT

All animal experimental procedures were approved by the Animal Care and Use Committee of Jiangsu University (UJS‐ACUC‐2025092801). Human serum sample collection was approved by Jinling Hospital's ethics committee board (approval number 2022DZGZR‐068).

## Supporting information


**Figure S1.** Gene Ontology (GO) enrichment analysis of the top 100 targets of hcmv‐miR‐UL36‐3p.

**Table S1.** Top 100 target genes of hcmv‐miR‐UL36‐3p predicted by TargetScan.

**Table S2.** miRNA and siRNA sequences.
