# Supplementary figures and images for: The HCMV‐encoded miR‐UL36‐3p promotes angiogenesis of endothelial cells by downregulating FOXO3
Source: Animal Model Exp Med. 2026 Mar 27;9(5):980–90. doi: 10.1002/ame2.70196 (PMC13331559; doi:10.1002/ame2.70196)

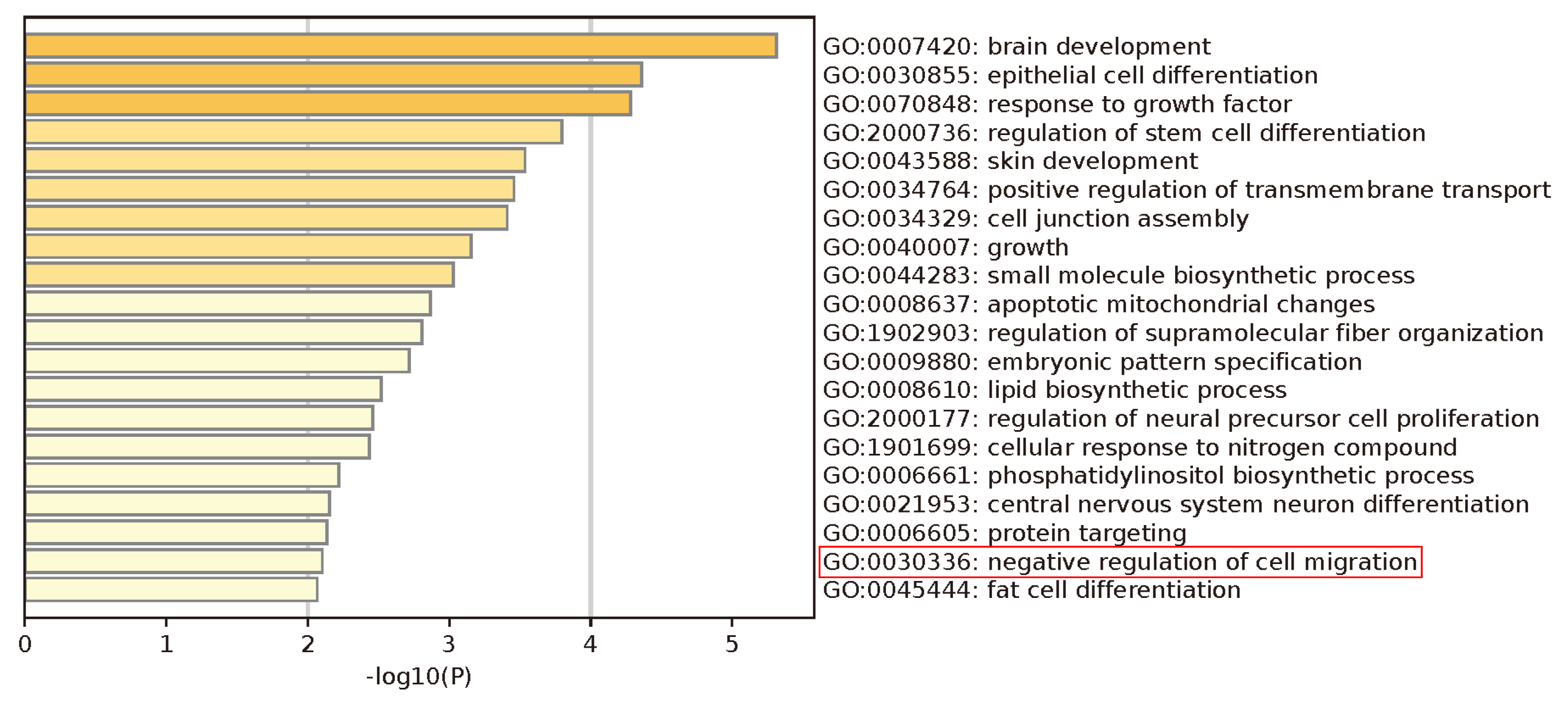

Supplement: Supplementary file 1 — Figure S1. Gene Ontology (GO) enrichment analysis of the top 100 targets of hcmv‐miR‐UL36‐3p. [file AME2-9-980-s003.tif]
